# A New Triterpene Hexaglycoside from the Bark of *Kalopanax septemlobus* (Thunb.) Koidz

**DOI:** 10.3390/molecules14114497

**Published:** 2009-11-09

**Authors:** Li-Shu Wang, Da-Qing Zhao, Tun-Hai Xu, Xue-Feng Zhou, Xian-Wen Yang, Yong-Hong Liu

**Affiliations:** 1 Jilin Provincial Academy of Chinese Medicine Sciences, Changchun 130021, China; E-Mail: wanglishu6856@yahoo.cn (L.W.); 2 Key Laboratory of Marine Bio-resources Sustainable Utilization, South China Sea Institute of Oceanology, Chinese Academy of Sciences, No. 164 West Xingang Road, Guangzhou 510-301, China; E-Mails: zhou-xuefeng@hotmail.com (X-F.Z.); yangxw76@163.com (X.Y.); 3 College of Pharmacy, Changchun University of Chinese Medicine, Changchun 130117, China; 4 School of Chinese Materia Medica, Beijing University of Chinese Medicine, Beijing 100102, China; E-Mail: thxu16@hotmail.com (T.X.)

**Keywords:** *Kalopanax septemlobus*, triterpene glycoside, Kalopanax-saponin, hederagenin

## Abstract

The new triterpene glycoside 3-*O*-β-D-xylopyranosyl-(1→4)-β-D-xylopyranosyl-(1→3)-α-L-rhamnopyranosyl-(1→2)-α-L-arabinopyranosylhederagenin 28-*O*-β-D-gluco-pyranosyl-(1→6)-β-D-glucopyranoside, named septemoside A (**1**), and the known 3-*O*-α-L-rhamnopyranosyl-(1→2)-*O*-α-L-arabinopyranoside-28-*O*-β-D-glucopyran-osyl-(1→6)-*O*-β-D-glucopyranosyl ester of hederagenin (**2**), were isolated from the bark of *Kalopanax septemlobus*. The structure elucidation of the compounds was based on spectroscopic evidence, including HRESIMS, 1D and 2D-NMR analysis.

## Introduction

*Kalopanax septemlobus* (Thunb.) Koidz. (= *K. septemlobum*) has been traditionally used for the treatment of rheumatic arthritis, nephritis edema, cholera, dysentery, and neurotic pain in China [1]. Kalopanax saponins A and B [2,3], septemloside I [4], septemloside III [5], kalopanax saponin C [6] liriodendrin, kalopanax saponin H [7], glycosides A, B, C, G_1_, I_2_, and J [8] were isolated from the bark of this plant. Kalopanax saponins C-F [9], kalopanax saponins G and H, clematis prosapogenin Cp7a [10] were isolated from the roots, and kalopanax saponins La, Lb, and Lc [11] isolated from the leaves. Glycosides A, B, C, F, G_1_, G_2_, I_2_, H, and J [12], E, K, and L [13], D_2_, I_1_, and K_1_ [14], glycosides C_2_, E_1_, F_2_, and G [15] were isolated from leaves of *K. septemlobum var*. *maximowichii.* Glycosides A, C, D, E, I, J, B_1_, B_2_, F_1_, F_2_, F_3_, G_1_, G_2_, G_3_, H_1_, and H_2 _ [16], glycosides B, C_1_, C_2_, E_1_, E_2_, F_1_, F_2_, H_3_, I_2_, I_1_, and K [15] were isolated from leaves of *K. septemlobum* *var typicum.* Glycoside B, kalopanax saponin A, saponin pg, tauroside G_2_, hederasaponin B, glycoside I_2_, hederoside A_2_, clemontanoside E, and hederoside H_2_ were isolated from the fruits of *K. septemlobum* *var typicum* [17].

The ethanolic extract of the stem bark of *K. septemlobus* exhibited significant hypoglycemic effects [7]. A lignan derivative, (-)-(7*R*, 8*S*)-dihydrodehydrodiconiferyl alcohol, isolated from *K. septemlobus* demonstrated neuritogenic activity, causing a marked induction of neurite outgrowth and an enhancement of nerve growth factor (NGF)-mediated neurite outgrowth from PC12 cells [18].

Our investigation on the constituents of the ethanol extract of the plant, led to the isolation of the new triterpenoidal saponin septemoside A (**1**), along with a known 3-*O*-α-L-rhamnopyranosyl-(1→2)-*O*-α-L-arabinopyranoside-28-*O*-β-D-glucopyranosyl-(1→6)-*O*-β-D-glucopyranosyl ester of heder-agenin (**2**) [15]. Herein, we describe the isolation and structure elucidation of **1**.

## Results and Discussion

Compound **1 **was obtained as white amorphous powder. The molecular formula of **1 **was determined as C_63_H_102_O_30_ on the basis of HRESIMS ([M+H]^+^, 1339.6539, calcd. for 1339.6534). The^ 1^H-NMR spectrum showed signals for six tertiary methyl groups at δ 1.06 (s), 1.03 (s), 0.99 (s), 0.86 (s), 0.76 (s), and 0.74 (s), and a secondary methyl group at δ 1.57 (d, *J* = 6.0 Hz), which correlated in the HMQC spectrum with seven carbons at δ_C_ 26.2, 14.3, 17.6, 16.3, 23.8, 33.2, and 18.6. The ^1^H-NMR spectrum also showed the presence of an olefinic proton resonance appearing as a distorted triplet at δ 5.27, characteristic for H-12 in pentacyclic triterpenes. The occurrence of the olefinic carbon signals at δ 123.1 and 144.2, corresponding to methine and quaternary carbons, suggested the presence of an endocyclic double bond at the 12-position in an oleanane skeleton. The ^13^C-NMR spectrum of **1 **exhibited sixty-three carbon resonances, thirty-three of which were in good agreement with the data reported for the oleanane triterpenoid hederagenin [5]. The remaining thirty carbon signals indicated the presence of six sugar units in the molecule, which was corroborated by the HMQC correlations displayed by carbons at δ 101.4, 95.8, 102.9, 107.8, 104.9, and 105.0, with anomeric protons at δ 6.23 (s), 6.12 (d, *J* = 8.0 Hz), 5.73 (s), 5.23 (d, *J* = 7.6 Hz), 4.94 (d, *J* = 6.4 Hz), and 4.86 (d, *J* = 8.0 Hz), respectively.

The acid hydrolysis of **1 **yielded hederagenin [5], and a sugar mixture consisting of D-xylose, D-glucose, L-rhamnose, and L-arabinose, which were identified by TLC and HPLC using authentic samples as references, as well as from the analysis of the ^1^H- and ^13^C-NMR chemical shifts. The alkaline hydrolysis of **1 **only yielded D-glucose, which was identified by comparison of its TLC and NMR spectra, with those of an authentic sample. The β configuration of the glucose unit was inferred from the coupling constant (*J* = 8.0 Hz) of the anomeric proton, and the chemical shift of the anomeric carbon (δ_C_ 95.8).

**Figure 1:**
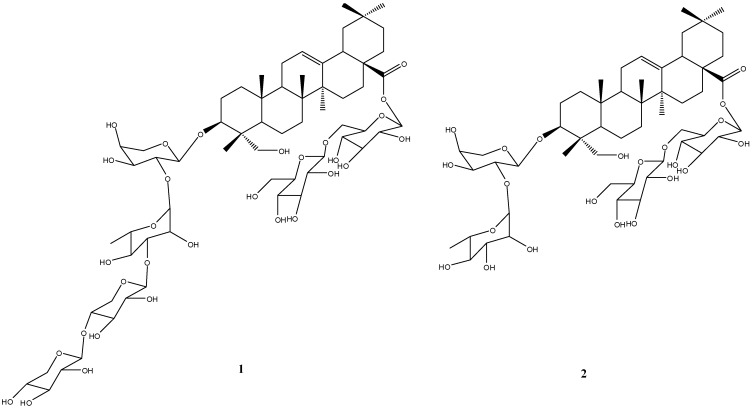
Structures of compounds **1** and **2**.

**Figure 2:**
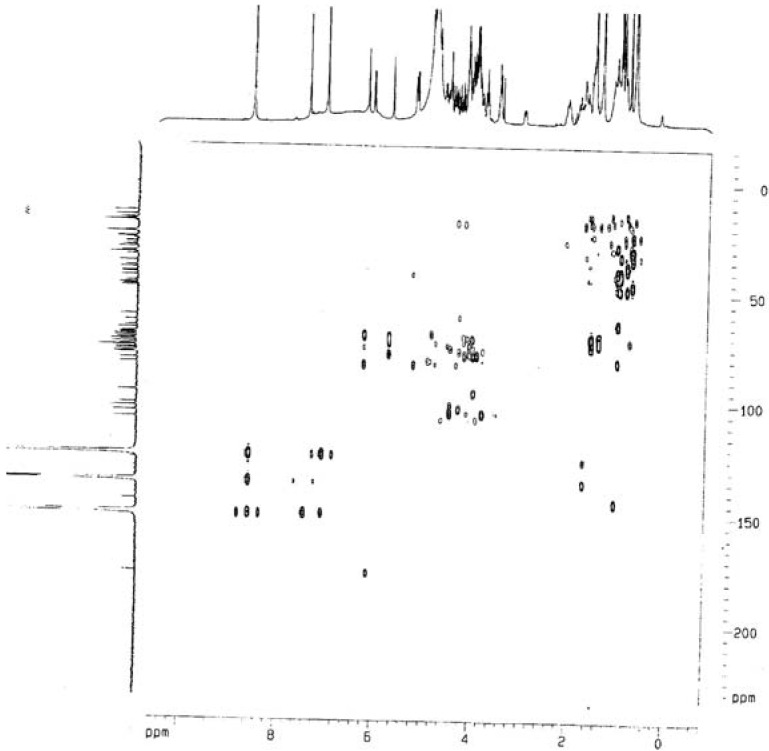
HMBC spectrum of compound **1**.

Evaluation of *J*_HH_ couplings allowed the identification of one α-L-arabinopyranosyl unit, one α-L-rhamno-pyranosyl, two β-D-glucopyranosyl, and two β-D-xylopyranosyl units. The protons of the monosaccharide residues were assigned starting from the anomeric protons by means of TOCSY experiment. On the basis of extensive 2D NMR experiments, along with the ESI-MS fragmentation pathway which displayed main fragment ions at *m/z* 326 attributable to a bissaccharide [glc + glc]^+^, 679 attributable to [aglycone + glc + Na]^+^, 905 attributable to [aglycone + ara + rha + xyl]^+^, 927 attributable to [aglycone + ara + rha + xyl + Na]^+^, and 943 attributable to [aglycone + ara + rha + xyl + K]^+^, the structure of **1 **suggested a bisdesmosidic saponin with a tetrasacharide linked at C-3 (δ 81.2), and a disaccharide linked at C-28 (δ 176.6) through an ester bond (Figure 1). This was further confirmed by the HMBC experiment (Figure 2). The identities of the sugar units linked to C-3 and C-28 were confirmed as L-arabinose and D-glucose, respectively, from the HMBC correlations of the anomeric protons at δ 4.86 and δ 6.12, with carbons at δ 81.2 and δ 176.6, respectively. Thus, the new structure of **1 **was determined as 3-*O*-β-D-xylopyranosyl-(1→4)-β-D-xylopyranosyl-(1→3)-α-L-rhamnopyranosyl-(1→2)-α-L-arabinopyranosylhederagenin 28-*O*-β-D-glucopyranosyl- (1→6)-β-D-glucopyranoside.

Compound **2** was isolated first time from the barks of this plant. Compounds **1 **and **2 **were evaluated for cytotoxicity by the MTT method, and showed a marginal activity against the human tumor cell lines HepG2 (hepatoma carcinoma), A549 (lung carcinoma), and Hela (cervical cancer).

**Table 1:** ^1^H-NMR and ^13^C-NMR spectral data of compound **1 **(recorded at 400/100 MHz in Pridine-d_5_; δ in ppm, *J* in Hz).

No.	^13^C	^1^H (*J* in Hz)	No.	^13^C	^1^H (*J* in Hz)
1	39.2	1.42 m, 0.95 m	C-3		
2	26.3	2.02 m, 1.03 m	Ara-1	105.0	4.86 d 8.0
3	81.2	4.16 m	2	76.7	4.06 m
4	43.6		3	75.5	3.90 m
5	47.9	1.63 m	4	69.7	4.19 m
6	19.2	1.55 m, 1.43 m	5	66.4	4.18 m, 3.64 m
7	32.9	1.71 m, 1.62 m	Rha-1	101.4	6.23 s
8	40.0		2	72.32	4.79 m
9	48.3	1.62 m	3	83.1	4.65 d 9.2
10	37.0		4	73.2	4.35 m
11	23.5	1.81 m, 1.76 m	5	69.9	4.55 m
12	123.1	5.27 brs	6	18.7	1.57 d 6.0
13	144.2		Xyl-1	107.8	5.23 d 7.6
14	42.3		2	75.8	3.80 m
15	28.4	2.03 m, 0.98 m	3	75.2	4.05 m
16	24.0	1.95 m, 1.89 m	4	74.1	4.19 m
17	47.2		5	64.1	4.22 m, 3.56 m
18	41.8	3.04 d 11.6, 4.0	Xyl-1	104.9	4.94 d 6.4
19	46.3	1.59 m, 1.45 m	2	74.1	3.98 m
20	30.9		3	77.3	3.55 m
21	34.1	1.18 m, 0.93 m	4	71.3	4.08 m
22	33.2	1.55 m, 1.07 m	5	67.6	4.15 m, 3.57 m
23	64.1	4.18 m, 3.80 t 8.4	C-28		
24	14.3	1.03 s	Glc-1	95.8	6.12 d 8.0
25	16.3	0.86 s	2	74.0	4.03 m
26	17.6	0.99 s	3	78.6	4.05 m
27	26.2	1.06 s	4	70.4	4.81 m
28	176.6		5	78.4	4.29 m
29	33.2	0.74 s	6	69.4	4.55 m, 4.22 m
30	23.8	0.76 s	Glc-1	102.9	5.73 s
			2	72.9	4.05 m
			3	78.9	4.03 m
			4	71.0	4.19 m
			5	78.2	4.05 m
			6	61.5	4.11 m, 4.01 m

## Experimental

### General

NMR spectra were recorded on a Bruker 400 NMR spectrometer with TMS as an internal standard. ESI-MS data were measured on a Agilent 1200 LC-MS spectrometer. HRESIMS and HRFAB-MS were obtained on Bruker Daltonics APEX II 47e and MAT 95XP (Thermo) mass spectrometers, respectively. The silica gel GF254 used for TLC were supplied by the Qingdao Marine Chemical Factory, Qingdao, China. Analytical HPLC was performed on a Hitachi L-2400 HPLC system, using a YMC ODS-H80 column (250 × 4.6 mm i.d., 4 μm) coupled to an Alltech ELSD 800 detector; semi-preparative HPLC was performed on a Hitachi L-2400 HPLC system, using a YMC ODS-H80 column (250 × 10 mm i.d., 4 μm) coupled to an Alltech ELSD 800 detector with flow-splitter valve (Parker: NS) set at a split ratio of 20:1 (collector: detector). Spots were detected on TLC under UV light or by heating after spraying with 5% H_2_SO_4_ in EtOH (v/v).

### Plant material

The barks of *Kalopanax septemlobus* were collected in Ji’an County, Jilin province, China, in June 2007, and identified as *K. septemlobus* with the assistance of Professor M. L. Deng of Department of Pharmacy, Changchun University of Chinese Medicine, China, The voucher specimen (No. 200706006) was deposited at Key Laboratory of Marine Bio-resources Sustainable Utilization, South China Sea Institute of Oceanology, Chinese Academy of Sciences. 

### Extraction and isolation

The dried barks (11 kg) were ground and extracted three times with aqueous 80% EtOH (30 L) under reflux. The combined EtOH extract was concentrated under vacuum, and then partitioned between petroleum ether and H_2_O, followed EtOAc, BuOH, to afford petroleum ether (29.3 g), EtOAc (113.4 g), and BuOH (163.4g) residues. The BuOH extract passed through a D101 macroporous resin column, then eluted with water, 30% EtOH, and 70% EtOH. The 70% EtOH eluate was collected and submitted to a D941 macroporous resin column to get the total saponins (31 g), which were chromatographed on Silica column eluted with CHCl_3_/MeOH/H_2_O (65:35:10, 55:45:10, and 45:55:10, lower layers) to give ten fractions. Fraction 7 (1.5 g) was chromatographed on ODS column eluted with MeOH/H_2_O (60:40) to give three fractions. Fraction 7-1 (250 mg) was further purified by reverse-phase HPLC (ODS, MeOH/H_2_O, 60:40) to yield compounds **1** (6.9 mg) and **2** (10.5 mg).

### Acid hydrolysis of **1**

A solution of **1** (3.0 mg) in 2.0 M trifluoroacetic acid (2 mL) was heated at 110 °C for 6 h (kept sealed). After cooling, the reaction mixture was extracted with EtOAc saturated with H_2_O (3 mL × 2). The EtOAc extract was evaporated to yield an aglycon, which was identified as hederagenin by comparison of spectroscopic and physical data with the reported values in the literature [5]. The H_2_O residue was concentrated under reduced pressure to yield a mixture of D-glucose, D-xylose, L-rhamnose, and L-arabinose which were identified by TLC and HPLC using authentic samples as references, as well as from the analysis of NMR spectrum [19].

### Alkaline hydrolysis of **1**

A solution of **1** (2.0 mg) in 5% NaOH (2 mL) was heated at 100 °C for 4 h (kept sealed). After cooling, the reaction mixture was evaporated under vacuum, then distilled water was added and the mixture extracted with BuOH saturated with H_2_O (3 mL × 3). The BuOH extract was evaporated to yield an aglycon, which was identified as hederagenin by comparison of spectroscopic and physical data with the reported values in the literature [5]. The H_2_O residue was concentrated under reduced pressure, yielding D-glucose, which was identified by comparison of its TLC and NMR spectra, with those of an authentic sample. 

### Characterization of septemoside A (**1**)

White amorphous solid; mp 230-231 °C; [α]_D_^25 ^= – 28.1 (c = 0.1, MeOH); HRESIMS ([M+H]^+^, m/z 1339.6539, calcd. for 1339.6534)); ^1^H-NMR and ^13^C-NMR spectral data: see Table 1.

## Conclusions

A phytochemical investigation on the bark of *Kalopanax septemlobus* led to the isolation of the new triterpene glycoside **1** and the known one **2**. Compound **2** was isolated from the bark of this plant for the first time. Compounds **1 **and **2 **showed marginal activity against the human tumor cell lines HepG2, A549, and Hela cell lines.
